# Supplementary material for: Validation of a Novel Predictive Algorithm for Kidney Failure in Patients Suffering from Chronic Kidney Disease: The Prognostic Reasoning System for Chronic Kidney Disease (PROGRES-CKD)
Source: Int J Environ Res Public Health. 2021 Nov 30;18(23):12649. doi: 10.3390/ijerph182312649 (PMC8656741; doi:10.3390/ijerph182312649)
Supplement: Supplementary file 1 [file ijerph-18-12649-s001.zip › ijerph-1461005-supplementary.pdf]

Supplementary material

Validation of a novel predictive algorithm for kidney failure in patients suffering from chronic kidney disease: The Prognostic Reasoning System for Chronic Kidney Disease (PROGRES-CKD).

Table of Contents

Supplementary Table S1. List of ICD10 codes used to abstract comorbidity variables **2**

Supplementary Table S2. Proteinuria Conversion table **3**

Supplementary results—Case study **4**

Supplementary Figure S1. Graphical output of PROGRES-CKD **5**

Supplementary Table S1. List of ICD10 codes used to abstract comorbidity variables

Cerebrovascular disease: G45-G46.9, H34.0, I60-I69.9

Chronic Pulmonary Disease: I27.8, I27.9, J40-J47.9, J60-J67.9, J68.4, J70.1, J70.3

Congestive heart failure: I09.9, I11.0, I13.0, I13.2, I25.5, I42.0, I42.5-I42.9, I43, I50, P29.0

Connective tissue disorder: M05-M06.9, M31.5, M32-M34.9, M35.1, M35.3, M36.0

Coronary artery disease: I21-I22.9, I25.2

Dementia: F00, F03.9, F05.1, G30, G31.1

Diabetes With Organ Damage: E10.2-E10.5, E10.7, E11.2, E11.5, E11.7, E12.2-E12.5, E12.7, E13.2-E13.5, E13.7, E14.2-E14.5, E14.7

Diabetes Without Complication: E10.0, E10.1, E10.6, E10.8, E10.9, E11.0, E11.1, E11.6, E11.8, E11.9, E12.0, E12.1, E12.6, E12.8, E12.9, E13.0, E13.1, E13.6, E13.8, E13.9, E14.0, E14.1, E14.6, E14.8, E14.9

Hemiplegia: G04.1, G11.4, G80.1, G80.2, G81-G82.9, G83.0-G83.4, G83.9

Hypertension: active antihypertensive drugs prescription (RAAS, Diuretics, Ca blocking, Beta blocking, Antiadrenergic) or mean(systolic)>130 or mean(diastolic)>90 in the last 12 months

Mild Liver Disease: B18, K70-K70.3, K70.9, K71.3-K71.5, K71.7, K73-K74, K76.0, K76.2, K76.4, K76.8, K76.9, Z94.4

Moderate Or Severe Liver Disease: I85, I86.4, I98.2, K70.4, K71.1, K72, K76.5-K76.7

Peripheral vascular disease: I70-I71, I73.1, I73.8, I77.1, I79.0, I79.2, K55.1, K55.8, K55.9, P29.0, Z95.8, Z95.9

Supplementary Table S2. Proteinuria Conversion table

ACR (mg/mmol)	Proteinuria (g/24 hrs)
≤ 3.5	< 0.15
3.6 – 29	0.150 – 0.499
30 – 69	0. 5 – 0.99
70 -150	1.0 – 1.5
151 – 450	1.5 – 4.5
> 450	> 4.5

Modified and Adapted from Lamb E, Mackenzie F, et al., Annals of Clinical Biochemistry 2009; 46: 205–217

Supplementary results—Case study

The patient was 72 years old, male, obese (BMI=34), suffering from stage 3a CKD (eGFR=55) caused by diabetes, never smoker. Known comorbidities were hypertension, chronic heart failure, cerebrovascular disease and coronary artery disease. He reported no hospitalizations over the 12 months before visit. Blood biomarkers were: serum albumin=4.40 g/dl; calcium=9.34 mg/dl; hemoglobin=12.53 g/dl; phosphate=3.26 mg/dl; proteinuria=0.39 g/24h; sodium=143 mmol/l.

Renal-ProScore24 predicted a low risk (15%). The final output of the predictive model for this patient is shown in Figure S1. In fact, this patient did not develop RRT within 24 months. Impact Analysis indicated the following variables as the most influential factors increasing the risk of KF within 24 months: presence of cerebrovascular disease (NL=1.79), sodium level (NL=1.67), obesity (NL=1.50), CHF (NL=1.48), presence of complicated diabetes (NL=1.35). Conversely, relative high eGFR (NL=0.36), low proteinuria (NL=0.59), calcium (NL=0.67), haemoglobin (NL=0.75), and advanced age (NL=0.87) favored the hypothesis that the patients would not initiate RRT within the prediction horizon of 24 months. VOI suggests that assessment of PTH concentration and rate of eGFR change would improve prognostic accuracy for this particular case by 3% and 2%, respectively. If the patient had both high PTH and steep eGFR change, his risk would be two-fold higher (joint Relative Risk=1.96) compared to the same patient without these risk factors.

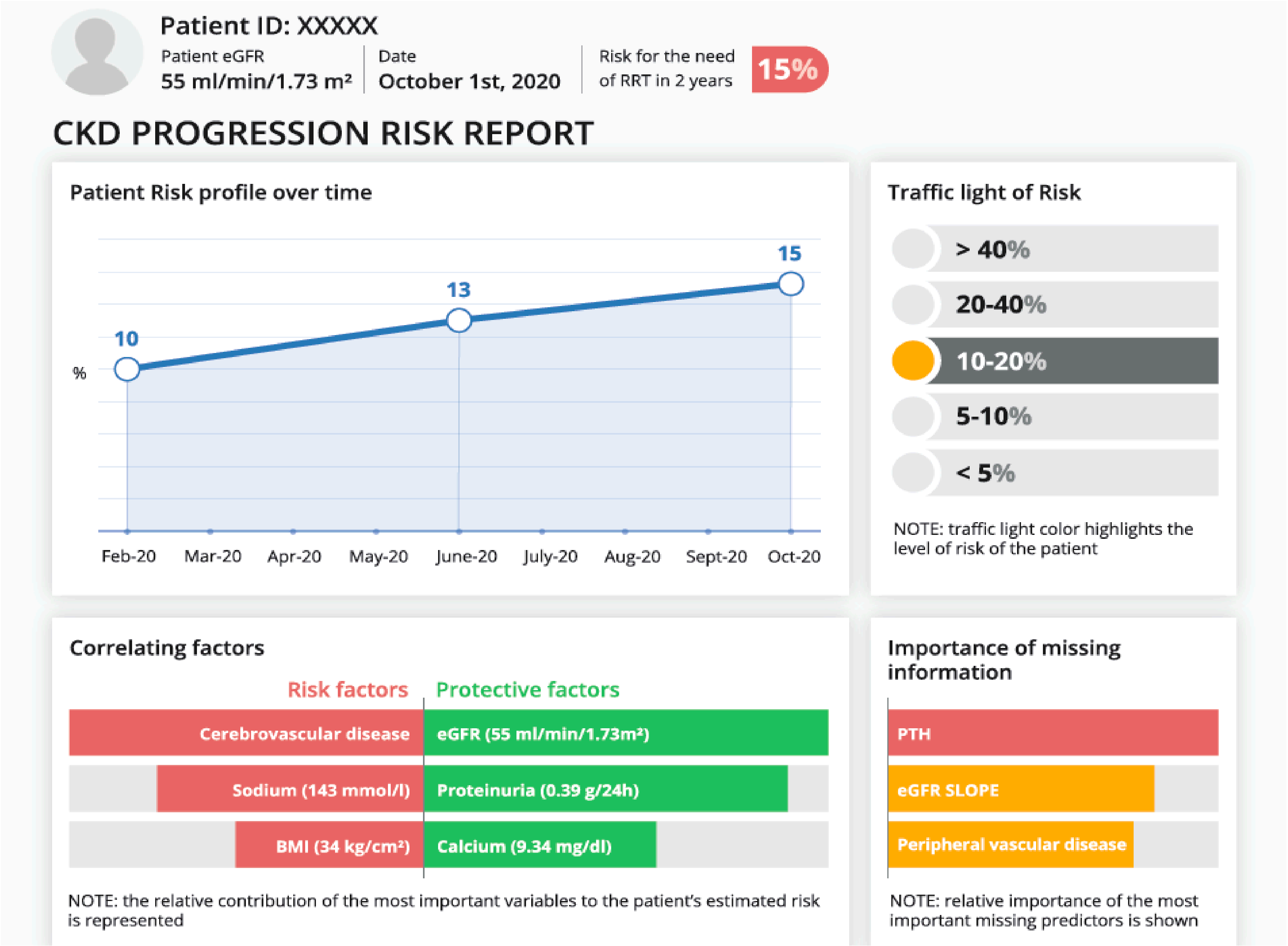

Supplementary Figure S1. Graphical output of PROGRES-CKD
